# Burnout in medical students and its psychological correlates with mentorship, motivation and professional values

**DOI:** 10.3389/fmed.2026.1752508

**Published:** 2026-02-02

**Authors:** Jane Tze Yn Lim, Kong Heng Lee, Ming Huey Siw, Farah Dina Khansa, Farah Alysha Mohd Farid Ridzwan Lee, Luke Sy-Cherng Woon, Suriati Mohamed Saini

**Affiliations:** 1 Department of Psychiatry, Faculty of Medicine, Universiti Kebangsaan Malaysia, Kuala Lumpur, Malaysia; 2 Hospital Canselor Tuanku Muhriz, Jalan Yaacob Latif, Bandar Tun Razak, Kuala Lumpur, Malaysia

**Keywords:** burnout, medical students, mentorship, motivation, professional values, professionalism, stress

## Abstract

**Introduction:**

Burnout is prevalent among medical students, arising from rigorous training and personal pressures. It is closely linked to stress-related and mood disorders. This study examined the prevalence of burnout among medical students at Universiti Kebangsaan Malaysia (UKM) and explored its association with mentorship, motivation and professional values, with the aim of informing strategies for mental health and educational support.

**Materials and methods:**

This cross-sectional study involved convenience sampling of 328 UKM medical students participating in UKM mentoring programmes. Structured questionnaires assessed sociodemographic characteristics, burnout (Copenhagen Burnout Inventory), mentorship experiences, motivation (Strength of Motivation for Medical School-Revised) and professional values (Physician Values in Practice Scale). Statistical analyses included Student’s *t*-test, chi-square test and logistic regression.

**Results:**

Among 328 respondents, high personal, work-related and client-related burnout rates were 60.4, 47.3 and 32.0%, respectively. Mentor approachability in lecturer mentorship was associated with lower work-related burnout (*p* < 0.001, OR = 0.62, 95% CI = 0.47–0.82). In peer mentorship, personal attributes were associated with lower personal (*p* < 0.001, OR = 0.41, 95% CI = 0.25–0.66) and work-related burnout (*p* = 0.013, OR = 0.70, 95% CI = 0.52–0.93). Persistence in motivation was associated with lower work-related (*p* < 0.001, OR = 0.85, 95% CI = 0.80–0.92) and client-related burnout (*p* < 0.001, OR = 0.82, 95% CI = 0.77–0.87). Professional values of service were linked to reduced client-related burnout (*p =* 0.009).

**Conclusion:**

Interventions for burnout among medical students should focus on enhancing the quality of lecturer and peer mentorship while also strengthening students’ motivation and professional values during medical training. Mental health educators play an essential role in embedding well-being strategies within medical education to reduce burnout.

## 1 Introduction

Burnout among students, particularly medical students, has become a growing concern due to the high-stress and demanding nature of medical programmes, and it is closely linked to stress-related and mood disorders. It is characterised by profound loss of interest in academic pursuits, diminished motivation and chronic exhaustion (1). In medical students, burnout can be conceptualised across three domains, namely personal burnout, work-related burnout and client-related burnout. Personal burnout reflects overall physical and psychological fatigue experienced in daily life (2, 3). Work-related burnout refers to exhaustion attributed to academic tasks such as lectures, studying, examinations and clinical responsibilities (4, 5). Client-related burnout describes fatigue associated with interactions with patients, peers or lecturers in their student role (6). A meta-analysis reported a high prevalence of burnout (44.2%) among medical students, with emotional exhaustion (40.8%), depersonalisation (35.1%) and reduced personal accomplishment (27.4%) being the most common symptoms (7).

Medical education presents multiple stressors. Academic demands often require prolonged study hours, leading to psychological distress and sleep disturbances (8). In addition, financial pressures, particularly among students from lower-income backgrounds, add another layer of stress (9). Work-life conflicts, including negative interactions with faculty or peers and a lack of social support, can intensify feelings of isolation and emotional dysregulation (10). The combination of intense academic demands, a competitive environment and exposure to patients’ suffering contribute to high burnout rates among medical students globally and locally (11, 12). In the Malaysian context, burnout rates among medical students vary across institutions, ranging from 10.1% in Selangor to 67.9% in Kelantan, with female students disproportionately affected and demonstrating greater psychiatric vulnerability (2, 11).

The impact of burnout among medical students is far-reaching, affecting individuals, institutions and society. At the individual level, burnout exerts a significant emotional toll. This can lead to disengagement from academic responsibilities and increased absenteeism. It is also associated with a spectrum of adverse outcomes, including diminished concentration, heightened anxiety, depression, substance misuse and even suicidal ideation (12–15). From a psychiatric perspective, these outcomes overlap with major depressive disorder (MDD), anxiety disorders, adjustment disorders and suicidal behaviour, underscoring burnout as a clinically relevant syndrome rather than a purely educational problem. At the institutional level, burnout imposes substantial economic burdens related to psychological support services and potential financial losses from student attrition, ultimately threatening the sustainability of the healthcare workforce (16–18). At the societal level, persistent burnout may undermine clinical competence and compromise future patient care (12, 13, 19).

Various studies highlight cultural and regional differences in coping strategies, ranging from alcohol consumption and self-medication to family support, across Europe, the Americas and Asia (20–25). While some of these strategies may exacerbate burnout, others, such as involvement in extracurricular activities and strong social support systems, have been shown to mitigate it (26). Individual-focused mental health services, such as counselling, mindfulness interventions and psychological support, are increasingly available within medical institutions (27, 28). Nevertheless, a critical gap remains regarding the role of medical education in addressing burnout as a psychiatric and public mental health concern. This underscores the need for systemic strategies to foster resilience. In this regard, mental health educators, including psychiatrists and psychologists, play a central role not only in clinical management but also in shaping medical education policies. By contributing to curriculum development, mental health educators can help integrate mental health literacy and structured mentorship frameworks into training programmes, thereby embedding preventive strategies within the medical curriculum.

The National University of Malaysia, more commonly known as Universiti Kebangsaan Malaysia (UKM), is a prominent public university with its Faculty of Medicine located in Kuala Lumpur. Established in 1972, the Faculty admits approximately 200–250 students annually, making it one of the largest medical programmes in Malaysia. UKM offers a dynamic learning environment, enriched by a diverse student body and comprehensive clinical training at its teaching hospital, Hospital Canselor Tuanku Muhriz (HCTM). Programmes aimed at professional development such as structured mentorship, motivational enhancement initiatives and the inculcation of professional values have been introduced to strengthen students’ resilience and psychological well-being.

UKM medical students represent an important population for studying burnout, as evidence indicates substantial psychological distress within this cohort. A recent study among clinical-year undergraduates at UKM found that more than one in five students met criteria for burnout during the COVID-19 period, with almost half reporting significant anxiety. Earlier work also demonstrated that first-year medical students experience considerable emotional strain, suggesting that burnout can emerge early and persist without targeted interventions (29).

UKM medical students benefit from both lecturer and peer mentorship programmes, which provide academic, personal and professional support. The details of each programme, including their structure, the roles of mentors and mechanisms for mental health support, are provided in the supplementary material.

Mentorship programmes function as protective mechanisms against burnout. Effective mentorship provides emotional support, academic guidance and professional development to help foster resilience and well-being, as well as to reduce psychological distress among medical students (30–32). It serves as a key personal and organisational resource that buffers the impact of heavy workload and emotional strain. Good mentors provide role modelling, guidance, feedback and emotional support, which is associated with higher engagement, better coping and lower burnout and distress among students and trainees. Conversely, absent, poor-quality or abusive mentorship can increase feelings of isolation, helplessness and unfairness, which are classic antecedents of cynicism and emotional exhaustion (8, 18, 33). Mentorship is recognised as a potential buffer against the development of stress-related and affective disorders by providing students with social connectedness, role modelling and early access to psychological support. Several mentorship models exist locally (34) and internationally (35), such as student-mentor pairings and clinical shadowing experiences, as exemplified by the University of Queensland, Australia (35). Involvement of mental health educators in mental health workshops, resilience training and crisis intervention planning positions them as key stakeholders in addressing burnout among medical students (36, 37).

Motivation is another key factor influencing burnout. According to Self-Determination Theory (SDT), autonomous motivation, which is learning driven by interest, personal values and choice, protects against burnout, whereas controlled or extrinsic motivation, driven by pressure, fear or external reward, predisposes students to it. Among medical students, higher intrinsic motivation is linked to greater engagement and lower emotional exhaustion, while high external pressure and fear of failure correlate with burnout, anxiety, and dropout intentions. Motivation additionally shapes stress appraisal: students with autonomous motivation are more likely to perceive demands as challenges rather than threats, lowering burnout risk (18, 33, 38).

Professional values, including altruism, compassion, integrity and commitment to patient welfare, provide meaning and purpose in medical training and are central to professional identity formation. When students’ professional values align with the learning environment, for example witnessing humane and ethical care role-modelled, they report a greater sense of meaning and lower burnout. Misalignment or moral distress, in contrast, is linked to cynicism, depersonalisation and emotional exhaustion, creating a negative cycle where burnout erodes professional values and empathy (8, 33, 39, 40).

Despite the increasing adoption of mentorship and professional development programmes aimed at instilling motivation and professional values among medical students, limited evidence exists regarding their impact on burnout rates and related psychiatric outcomes such as anxiety, depression and suicidal ideation. Hence, this study aims to examine the prevalence of burnout among UKM medical students and to investigate the influence of mentorship, motivation and professional values embedded within the medical curriculum on burnout levels. It is hypothesised that burnout is highly prevalent in this population and that mentorship, intrinsic motivation and professional values are significantly associated with burnout.

## 2 Materials and methods

### 2.1 Study population

This study targeted all undergraduate medical students at UKM involved in both lecturer and peer mentorship programmes. It employed a cross-sectional study design using a structured questionnaire as the primary data collection instrument. As peer mentors are typically assigned from Year 2 onwards, the study comprised students from Year 2 to Year 5 (N = 629). Sample size was calculated using the prevalence formula with finite population correction (2), based on a 67.9% prevalence rate (*p* = 0.68), precision (d) = 0.05 and Z = 1.96. The resulting sample size was 216. To account for non-responses and missing data, a 45% increase was applied, resulting in a final target sample size of 313.

### 2.2 Data collection

Data collection was conducted from 1 February 2024 to 30 June 2024. A weekly online survey link was distributed through group leaders and a QR code was made available before or after teaching sessions. Inclusion criteria were (i) involvement in both lecturer and peer mentorship programmes, (ii) provision of written consent and (iii) proficiency in English. Exclusion criteria were (i) involvement in only one mentorship programme due to year of entry into medical school, (ii) non-consent to respond to the questionnaire and (iii) student researchers conducting the study. Eligibility was assessed through sociodemographic screening embedded within the questionnaire. To ensure one response per individual, access was restricted to official university email logins. Anonymity was maintained, however, as no email data were stored.

### 2.3 Study tools

Data were collected using a structured, self-administered online questionnaire consisting of five sections: (i) sociodemographic information, (ii) burnout levels, (iii) mentorship experiences, (iv) strength of motivation and (v) professional values.

Burnout was assessed using the Copenhagen Burnout Inventory (CBI), which has been validated for local use. It comprises 19 items across three domains: personal, work-related (adapted to academic activities) and client-related (adapted to clinical interactions), rated on a 5-point Likert scale ranging from “always” to “never/almost never” (41). The CBI demonstrates high internal consistency (Cronbach’s alpha: personal = 0.87; work = 0.87; client = 0.85). A cut-off score of ≥50 was used to indicate high burnout, consistent with the original developers’ recommendations (42). This threshold has been applied in a few local studies among university and medical student populations, demonstrating that sizeable proportions exceed this level, suggesting a substantial burnout burden among Malaysian students (2, 3).

Lecturer mentorship for medical students was measured using a 25-item questionnaire adapted from Sparshadeep et al. (43), encompassing five domains: programme structure, mentor’s approachability, professional development, mentor’s attitude and future expectations. Responses were recorded on a 5-point Likert scale. The instrument demonstrated excellent internal consistency (Cronbach’s alpha = 0.97) (33).

Peer mentorship was assessed using a 12-item questionnaire developed by Mohd Shafiaai et al. (44), focusing on four domains: pedagogical skills, leadership, personal attributes and communication. The questionnaire was validated locally for use in medical students and employed a 5-point Likert scale ranging from ‘strongly agree’ to ‘strongly disagree’. It demonstrated acceptable internal consistency (Cronbach’s alpha = 0.797) (34).

Motivation in medical students was measured using the Strength of Motivation for Medical School-Revised (SMMS-R) scale (45), comprising 15 items across three subscales: willingness to sacrifice (*α* = 0.70), readiness to start (α = 0.67) and persistence (α = 0.55) (45, 46). Some items were reverse-scored, and responses were recorded on a six-point Likert scale. Despite the lower internal consistency of the persistence subscale, it was retained as a key factor in motivation because it taps students’ willingness to continue studying medicine despite difficulties, which is conceptually close to grit and academic resilience and is strongly linked to burnout and engagement in the literature (33).

Professional values were assessed using the Physician Values in Practice Scale (PVIPS), specifically the “Prestige” and “Service” subscales (47). The PVIPS has demonstrated strong construct validity and internal consistency (*α* = 0.85–0.86) and has been validated for use among medical students (47).

### 2.4 Statistical analysis

Data were analysed using IBM SPSS Statistics, version 29. Descriptive statistics (means, standard deviations, frequencies, and percentages) were used to summarise participant characteristics, burnout levels, mentorship measures, strength of motivation, and professional values. For bivariate analyses, Pearson’s chi-squared tests were applied to examine associations between categorical variables and burnout categories, while independent-samples Student’s *t*-tests were used to compare continuous variables across low and high burnout groups for each of the CBI dimensions.

The three CBI dimensions (personal burnout, work related burnout, and client related burnout) were treated as outcome variables in all inferential analyses. Sociodemographic characteristics, lecturer mentorship variables, peer mentorship variables, strength of motivation subscales and professional values were treated as independent variables. Student’s *t*-tests and Pearson’s chi-squared tests were used to examine univariate associations between independent variables and each burnout dimension. Binary logistic regression was then conducted to identify factors independently associated with high burnout for each CBI dimension, adjusting for relevant covariates. Statistical significance was set at *p* < 0.05 for all analyses.

## 3 Results

A total of 381 students responded to the questionnaires, of whom 53 were excluded based on the study criteria. Two respondents did not provide consent, and 51 participated only in the lecturer mentorship programme. This resulted in 328 valid responses. The characteristics of the study participants are presented in Table 1.

**Table 1:** Characteristics of study respondents (*n* = 328).

Variables	*n* (%)
Sex	
Male	91 (27.7%)
Female	237 (72.3%)
Ethnicity	
Malay	139 (42.4%)
Chinese	107 (32.6%)
Indian	63 (19.2%)
Others	19 (5.8%)
Year of study	
Year 2	111 (33.8%)
Year 3	93 (28.4%)
Year 4	72 (22%)
Year 5	52 (15.9%)
Extra-curricular activities	
Yes	223 (68%)
No	105 (32%)
Parent’s occupation	
Medical	54 (16.5%)
Non-medical	274 (83.5%)
Medical course against own interest	
Yes	71 (21.6%)
No	257 (78.4%)
Relationship status	
Single	257 (78.4%)
In a relationship	71 (21.6%)

The majority of respondents were female (72.3%), with the largest proportion from Year 2 (33.8%). Most participants were Malay (42.4%) and single (78.4%), while 68% reported participation in extracurricular activities. Approximately 16.5% had parents working in the medical field. Notably, 78.4% of respondents reported choosing medicine willingly, whereas 21.6% did so against their interest.

**Figure1:**
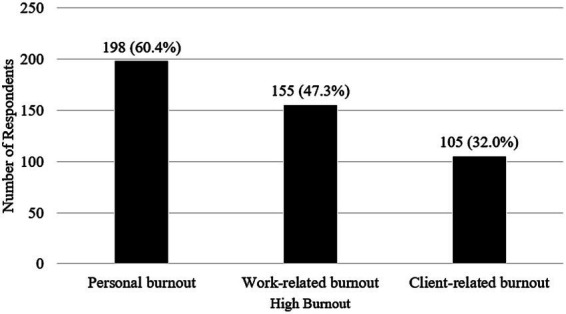
Number of respondents with high personal, work-related, and client-related burnout (CBI).

As shown in Figure 1, high personal burnout was reported in 198 of 328 students (60.4%), followed by high work-related burnout in 155 of 328 students (47.3%) and high client-related burnout in 105 of 328 students (32.0%).

Table 2 presents the levels of burnout according to sociodemographic characteristics and associated determinants. Female respondents reported significantly higher levels of personal (*p* = 0.048) and work-related burnout (*p* = 0.048). Client-related burnout was significantly associated with parental occupation, with students whose parents worked in non-medical fields showing higher burnout levels (*p =* 0.014).

**Table 2:** Burnout levels by sociodemographic characteristics and associated determinants across all CBI dimensions.

Variables	Personal burnout			Work-related burnout			Client-related burnout		
	Low	High	*p*-value	Low	High	*p*-value	Low	High	*p*-value
Sex *n* (%)			0.048*			0.048*			0.175
Male	45 (34.6%)	46 (23.2%)		56 (32.4%)	35 (22.6%)		67 (30.0%)	24 (22.9%)	
Female	85 (65.4%)	152 (76.8%)		117 (67.6%)	120 (77.4%)		156 (70.0%)	81 (77.1%)	
Ethnicity *n* (%)			0.496			0.748			0.607
Malay	49 (37.7%)	90 (45.5%)		69 (39.9%)	70 (45.2%)		89 (39.9%)	50 (47.6%)	
Chinese	48 (36.9%)	59 (29.8%)		60 (34.7%)	47 (30.3%)		76 (34.1%)	31 (29.5%)	
Indian	25 (19.2%)	38 (19.2%)		33 (19.1%)	30 (19.4%)		44 (19.7%)	19 (18.1%)	
Others	8 (6.2%)	11 (5.6%)		11 (6.4%)	8 (5.2%)		14 (6.3%)	5 (4.8%)	
Year of study *n* (%)			0.63			0.388			0.604
Year 2	45 (34.6%)	66 (33.3%)		60 (34.7%)	51 (32.9%)		75 (33.6%)	36 (34.3%)	
Year 3	41 (31.5%)	52 (26.3%)		53 (30.6%)	40 (25.8%)		68 (30.5%)	25 (23.8%)	
Year 4	26 (20.0%)	46 (23.2%)		38 (22.0%)	34 (21.9%)		46 (20.6%)	26 (24.8%)	
Year 5	18 (13.8%)	34 (17.2%)		22 (12.7%)	30 (19.4%)		34 (15.2%)	18 (17.1%)	
Extra-curricular activities *n* (%)			0.382			0.13			0.105
Yes	92 (70.8%)	131 (66.2%)		124 (71.7%)	99 (63.9%)		158 (70.9%)	65 (61.9%)	
No	38 (29.2%)	67 (33.8%)		49 (28.3%)	56 (36.1%)		65 (29.2%)	40 (38.1%)	
Parent’s occupation *n* (%)			0.273			0.877			0.014*
Medical	25 (19.2%)	29 (14.6%)		29 (16.8%)	25 (16.1%)		29 (13.0%)	25 (23.8%)	
Non-medical	105 (80.8%)	169 (85.4%)		144 (83.2%)	130 (83.9%)		194 (87.0%)	80 (76.2%)	
Against own interest *n* (%)			0.159			0.143			0.13
Yes	23 (17.7%)	48 (24.2%)		32 (18.5%)	39 (25.2%)		43 (19.3%)	28 (26.7%)	
No	107 (82.3%)	150 (75.8%)		141 (81.5%)	116 (74.8%)		180 (80.7%)	77 (73.3%)	
Relationship status *n* (%)			0.755			0.697			0.174
Single	103 (79.2%)	154 (77.8%)		137 (79.2%)	120 (77.4%)		220 (78.0%)	37 (80.4%)	
In a relationship	27 (20.8%)	44 (22.2%)		36 (20.8%)	35 (22.6%)		62 (22.0%)	9 (19.6%)	
Lecturer mentorship (μ ± SD)									
Mentoring programme	3.64 ± 0.852	3.50 ± 0.877	0.147	3.70 ± 0.851	3.40 ± 0.865	0.002*	3.57 ± 0.846	3.52 ± 0.919	0.561
Mentor’s approachability	3.89 ± 0.744	3.78 ± 0.861	0.21	3.97 ± 0.714	3.66 ± 0.894	< 0.001*	3.85 ± 0.790	3.76 ± 0.873	0.378
Student’s professional development	3.77 ± 0.871	3.64 ± 0.907	0.207	3.81 ± 0.854	3.56 ± 0.919	0.010*	3.71 ± 0.879	3.66 ± 0.927	0.659
Mentor’s attitude	4.03 ± 0.836	3.95 ± 0.858	0.404	4.12 ± 0.809	3.84 ± 0.870	0.002*	4.03 ± 0.830	3.89 ± 0.883	0.141
Future expectations	3.91 ± 0.838	3.85 ± 0.739	0.531	4.00 ± 0.805	3.73 ± 0.902	0.004*	3.89 ± 0.864	3.83 ± 0.862	0.571
Overall *n* (%)			0.227			< 0.001*			0.384
Effective	56 (43.1%)	74 (37.4%)		79 (45.7%)	51 (32.9%)		92 (41.3%)	38 (36.2%)	
Not effective	74 (56.9%)	124 (62.6%)		94 (54.3%)	104 (67.1%)		131 (58.7%)	67 (63.8%)	
Peer mentorship (μ ± SD)									
Communication	3.87 ± 0.726	3.80 ± 0.707	0.355	3.91 ± 0.681	3.74 ± 0.742	0.036*	3.87 ± 0.697	3.746 ± 0.748	0.148
Pedagogical	3.90 ± 0.696	3.71 ± 0.720	0.021*	3.88 ± 0.689	3.69 ± 0.733	0.016*	3.83 ± 0.716	3.71 ± 0.711	0.167
Personal Attributes	3.87 ± 0.740	3.59 ± 0.787	0.001*	3.81 ± 0.740	3.59 ± 0.809	0.012*	3.72 ± 0.784	3.66 ± 0.774	0.5
Leadership	3.87 ± 0.830	3.79 ± 0.834	0.387	3.90 ± 0.805	3.73 ± 0.855	0.060*	3.87 ± 0.836	3.71 ± 0.817	0.114
Overall	3.88 (0.703)	3.72 (0.684)	0.047*	3.87 ± 0.676	3.69 ± 0.705	0.015*	3.82 ± 0.692	3.71 ± 0.697	0.165
Strength of motivation (μ ± SD)									
Willingness to sacrifice	17.53 ± 3.269	17.00 ± 3.790	0.196	17.75 ± 3.264	16.61 ± 3.857	0.004*	17.61 ± 3.451	16.38 ± 3.771	0.004*
Readiness to start	16.47 ± 3.421	16.35 ± 4.009	0.778	16.64 ± 3.532	16.13 ± 4.038	0.226	16.68 ± 3.766	15.79 ± 3.764	0.046*
Persistence	18.66 ± 3.921	17.25 ± 4.507	0.004*	18.93 ± 3.895	16.55 ± 4.465	< 0.001*	18.90 ± 3.891	15.50 ± 4.335	< 0.001*
Overall *n* (%)			0.3			0.011*			< 0.001*
Low	2 (1.5%)	12 (6.1%)		4 (2.3%)	10 (6.5%)		5 (2.2%)	9 (8.6%)	
Moderate	99 (76.2%)	144 (72.7%)		124 (71.7%)	119 (76.8%)		161 (72.2%)	82 (78.1%)	
High	29 (22.3%)	42 (21.2%)		45 (26.0%)	26 (16.8%)		57 (25.6%)	14 (13.3%)	
Values in professionalism *n* (%)									
Prestige			0.96			0.433			0.383
Low	6 (4.6%)	8 (4.0%)		5 (2.9%)	9 (5.8%)		10 (4.5%)	4 (3.8%)	
Moderate	38 (29.2%)	65 (32.8%)		54 (31.2%)	49 (31.6%)		66 (29.6%)	37 (35.2%)	
High	86 (66.2%)	125 (63.1%)		114 (65.9%)	97 (62.6%)		147 (65.9%)	64 (61.0%)	
Service			0.331			0.26			0.009*
Low	3 (2.3%)	5 (2.5%)		3 (1.7%)	5 (3.2%)		3 (1.3%)	5 (4.8%)	
Moderate	33 (25.4%)	45 (22.7%)		39 (22.5%)	39 (25.2%)		48 (21.5%)	30 (28.6%)	
High	94 (72.3%)	148 (74.7%)		131 (75.7%)	111 (71.6%)		172 (77.1%)	70 (66.7%)	

*n* (%) = categorical variables; mean ± SD (mean = μ; SD = standard deviation) = continuous variables’, Pearson’s chi-squared test for categorical data, Student’s *t*-test for continuous data, *p < 0.05.

Overall, effective lecturer mentorship was significantly associated with lower work-related burnout (*p* < 0.001), while peer mentorship was significantly associated with lower personal (*p* = 0.047) and work-related burnout (*p* = 0.015). Strength of motivation was significantly associated with both work-related (*p* = 0.011) and client-related burnout (*p* < 0.001). Regarding professional values, the service subscale was significantly associated with lower client-related burnout (*p* = 0.009), whereas no significant association was found for the prestige subscale.

Both specific lecturer mentorship domains and overall lecturer mentorship were significantly associated with work-related burnout. Significant differences were observed for the mentoring programme (*p* = 0.002), mentor’s approachability (*p* < 0.001), professional development (*p* = 0.010), mentor’s attitude (*p* = 0.002), future expectations (*p* = 0.004) and the overall effectiveness of lecturer mentorship (*p* < 0.001). Peer mentorship showed significant associations with personal and work-related burnout, particularly for pedagogical attributes (*p* = 0.021 and *p* = 0.016, respectively) and personal attributes (*p* = 0.001 and *p* = 0.012, respectively), with overall peer mentorship scores also differing for personal (*p* = 0.047) and work-related burnout (*p* = 0.015).

Strength of motivation was significantly associated with all three burnout dimensions for the persistence subscale, with significant differences observed for personal burnout (*p* = 0.004), work-related burnout (*p* < 0.001) and client-related burnout (*p* < 0.001). Willingness to sacrifice was also significantly associated with work-related burnout (*p* = 0.004) and client-related burnout (*p* = 0.004). Regarding professional values, the service subscale was significantly associated with client-related burnout (*p* = 0.009), whereas the prestige subscale showed no significant associations.

Table 3 summarises factors associated with high burnout among respondents. Within the lecturer mentorship programme, mentor approachability emerged as a significant factor of lower work-related burnout (*p* < 0.001, OR = 0.62, 95% CI = 0.47–0.82). In the peer mentorship programme, personal attributes were significantly associated with lower personal burnout (*p* < 0.001, OR = 0.41, 95% CI = 0.25–0.66) and lower work-related burnout (*p* = 0.013, OR = 0.70, 95% CI = 0.52–0.93). For strength of motivation, the persistence subscale was significantly linked to lower work-related burnout (*p* < 0.001, OR = 0.85, 95% CI = 0.80–0.92) and lower client-related burnout (*p* < 0.001, OR = 0.82, 95% CI = 0.77–0.87).

**Table 3:** Factors associated with high burnout among study respondents.

Variable	Odds ratio	95% confidence interval	*p-*value
Personal burnout			
Peer mentorship			
Communication	1.78	1.07–2.95	0.026*
Pedagogical	0.609	0.25–1.50	0.282
Personal attributes	0.411	0.25–0.66	< 0.001*
Leadership	1.207	0.75–1.95	0.442
Work-related burnout			
Lecturer mentorship			
Mentoring programme	0.749	0.46–1.22	0.242
Mentor’s approachability	0.62	0.47–0.82	< 0.001*
Student’s professional development	1.239	0.76–2.01	0.388
Mentor’s attitude	0.841	0.58–1.21	0.352
Future expectations	0.961	0.56–1.66	0.886
Peer mentorship			
Communication	1.008	0.53–1.92	0.982
Pedagogical	0.854	0.47–1.54	0.601
Personal attributes	0.696	0.52–0.93	0.013*
Leadership	0.994	0.64–1.56	0.979
Strength of motivation			
Willingness to sacrifice	0.904	0.82–0.99	0.036*
Readiness to start	1.03	0.95–1.12	0.503
Persistence	0.853	0.80–0.92	< 0.001*
Client-related burnout			
Strength of motivation			
Willingness to sacrifice	0.916	0.83–1.02	0.102
Readiness to start	0.987	0.90–1.09	0.794
Persistence	0.819	0.77–0.87	< 0.001*

*p < 0.05.

## 4 Discussion

Our study found a high prevalence of burnout among medical students, with 60.4% experiencing personal burnout, 47.3% work-related burnout and 32% client-related burnout. These results are comparable to a study from Universiti Sains Malaysia (USM), which reported 81.6% of students experiencing personal burnout, 73.7% work-related burnout and 68.6% client-related burnout (2). In contrast, a study in Kazakhstan reported 28% of students experiencing burnout (48). A systematic review of 34 studies across 18 countries highlighted the wide variation in burnout prevalence, ranging from 8.9% in Saudi Arabia to 91.1% in Iran (49). Such disparities may be explained by differences in educational environments, cultural expectations and healthcare systems, which play important roles in shaping burnout levels among medical students (50). In addition, the observed prevalence may have been influenced by the availability of structured mentorship programmes at UKM and by the use of the Copenhagen Burnout Inventory (CBI) rather than other instruments such as the Maslach Burnout Inventory (MBI) or Oldenburg Burnout Inventory (OLBI).

Medical curriculum structure influences burnout with variations in academic workload, clinical exposure, and assessment methods in different institutions. In intensive academic environment like UKM and USM, burnout is more prevalent, similarly in curricula that prioritise rote memorisation over analytical and critical thinking (3). Future research should explore how curriculum design, learning environments and assessment intensity affect burnout and whether self-directed learning (SDL) approaches and formative assessments can help to alleviate stress (51).

Despite growing mental health awareness, many Malaysian medical schools still lack adequate resources to support students. The limited availability of counselling services and structured mental health programmes leaves students feeling isolated in their struggles with burnout (3, 52, 53). Stigma surrounding mental health further discourages help-seeking behaviours (49, 54). Without timely support, emotional strain may progress to depression, anxiety or even suicidal ideation, highlighting the need for proactive and accessible student well-being services. Mental health educators play a pivotal role in leading mental health campaigns, wellness programmes, early screening and timely interventions. By championing such initiatives, they can foster help-seeking behaviours, reduce stigma and mitigate burnout and its psychological consequences within the medical education system.

### 4.1 Sociodemographic factors

Our study confirmed that female students were more likely to experience personal and work-related burnout, consistent with other local studies (2, 3). This finding aligns with international research showing higher burnout prevalence among female medical students, often attributed to additional academic, societal and cultural pressures (14, 55, 56). The dual burden of academic responsibilities and societal expectations, particularly in patriarchal cultures, may contribute to this disparity. Female students are also more likely to adopt emotion-focused coping strategies, such as rumination, which increase vulnerability to burnout and depression, whereas male students more frequently use problem-focused strategies that are generally more effective for stress management (55, 56). Furthermore, reports indicate that up to 50% of female medical students experience sexual harassment, which may exacerbate psychological burden and increase the risk of depression and post-traumatic stress disorder (PTSD) (57). Such harassment has also been linked to academic disengagement and burnout (56). These findings underscore the need for trauma-sensitive and gender-aware mentorship, including flexible mentor–mentee matching and supportive institutional policies, to help mitigate gender-specific stressors and reduce burnout risk.

Parental occupation was significantly associated with burnout in our study, with students whose parents worked in non-medical fields reported higher levels of client-related burnout. This contrasts with prior research in collectivist cultures, which often emphasises that students with healthcare professional parents experience greater stress due to high parental expectations (58–61). One potential explanation is that students from medical families are often exposed earlier to clinical narratives, professional norms and coping strategies such as boundary setting or debriefing about difficult cases, which can normalise distress and reduce emotional shock when first encountering real patients (62, 63). In contrast, students from non-medical backgrounds may enter clinical settings with fewer concrete role models for managing suffering, conflict, or complaints, making these experiences feel more overwhelming or personally threatening (8, 64). Additionally, parental expectations and guidance may differ. Parents with medical experience can frame difficult patient interactions as expected professional challenges, whereas non-medical parents may focus more on grades or prestige, offering less tailored advice on coping with emotionally demanding situations (65, 66). According to Self-Determination Theory (SDT), autonomy, competence and relatedness are essential for psychological well-being (67). When these psychological needs are eroded, students may become more vulnerable to anxiety, low mood and maladaptive coping. This suggests that burnout often overlaps with broader mental health concerns, rather than being confined to academic performance. Mental health educators can mitigate these effects by introducing family-focused psychoeducation programmes aimed at aligning parental expectations with students’ mental health needs.

### 4.2 Lecturer mentorship

Lecturer mentorship helps reduce burnout, as evidenced in our findings. Students who view mentors as approachable report less academic burnout, consistent with findings from another qualitative study (43). Mentorship quality is often undermined by gender bias, poor communication, infrequent meetings and mentors’ blaming attitudes (43, 68, 69). Such negative dynamics weaken trust and discourage help-seeking, leading to increased stress, low self-esteem and burnout (69). Strengthening mentor-student relationships, particularly by enhancing approachability, may therefore reduce burnout. Improving mentor approachability can be facilitated through open communication and the creation of a supportive environment. Regular check-ins help to build trust and rapport, allowing students to feel safe in expressing concerns. Virtual meetings may also reduce barriers for students during out-of-campus postings (32). Empathy and emotional intelligence are central to effective mentorship, enabling students to feel heard and supported while reducing burnout (34). Mental health educators can enrich mentorship programmes by integrating emotional intelligence, empathy and reflective communication training, alongside personalised approaches like mentorship contracts and reflective sessions to foster resilience, leadership and coping skills, which are often absent from traditional clinical training (70).

### 4.3 Peer mentorship

Peer mentorship also reduced burnout, consistent with findings by Mohd Shafiaai et al., who demonstrated that personal attributes were strongly associated with lower personal and work-related burnout (44). Existing evidence suggests that peer and near-peer mentorship programmes are associated with reduced stress and burnout risk among medical students, mainly by lowering anxiety, enhancing coping and work-life balance, and strengthening a sense of connection rather than directly shifting burnout scores (30, 71). Structured programmes, where mentors are trained and meetings are regular, appear particularly effective in fostering community, belonging and professional identity, which are protective against burnout (72, 73). Peer mentorship helps students build organisational and time management skills while fostering competence and a sense of belonging, which reduce stress, prevent loneliness and enhance resilience (70). Mentorship provides emotional support, normalises difficulties, and facilitates help-seeking, addressing social isolation and lack of support, which are known correlates of burnout (18). Well-designed programmes with clear expectations and structured contact further enhance benefits for both mentees and mentors, supporting resilience and long-term sustainability (73, 74). Mental health educators could enhance peer mentorship programmes by supervising training workshops, teaching mental health first aid and ensuring clear referral pathways for at-risk students.

### 4.4 Motivation

Motivation emerged as a key protective factor against burnout. Students who demonstrated higher persistence reported significantly lower levels of client- and work-related burnout. This aligns with research showing that intrinsic motivation protects against burnout, while low or externally driven motivation is associated with higher burnout among medical students (75, 76). Intrinsic motivation correlates with lower emotional exhaustion and higher personal accomplishment, whereas low motivation predicts higher burnout even after controlling for other factors (10, 77). Within the framework of Self-Determination Theory (SDT), achievement motivation fosters a sense of purpose, resilience and effective coping (78–80). These effects operate through supporting autonomy, competence and relatedness, which help students perceive academic and clinical demands as meaningful challenges rather than draining pressures. Controlling environments that undermine these needs increase extrinsic motivation or amotivation, linked to higher burnout (81). Conversely, students with lower motivation may struggle to derive meaning from their responsibilities, making them more vulnerable to burnout. Curricula that strengthen intrinsic motivation through autonomy-supportive teaching, meaningful feedback, choice in learning activities and alignment with professional values can help prevent burnout. Monitoring students’ motivation alongside burnout measures may identify at-risk groups and guide targeted interventions (82, 83).

### 4.5 Professional values

Our study also highlighted that students with higher levels of commitment to professional values, particularly service, experienced lower client-related burnout. This suggests that integrating professional values into medical education could reduce emotional strain associated with patient interactions. Similar patterns have been observed in other research. At UKM, professional values are integrated into the curriculum through the Personal and Professional Advancement (PPA) module, which promotes skills such as effective communication, ethical practice, bedside manners, critical thinking and professional judgement. Beyond shaping professional conduct, these competencies foster emotional intelligence and self-reflection, both protective against stress and disengagement. Teaching strategies include lectures, reflective learning, role-play of real-life scenarios and outdoor camps, all designed to help students internalise and apply professional values.

To specifically cultivate the value of service, integrating service-learning programmes, which combine community engagement with academic objectives, can reduce client-related burnout while strengthening students’ sense of purpose and professional identity (84, 85). In such programmes, mental health educators play a role in linking psychiatric education with community engagement, promoting mental health awareness, social–emotional learning and resilience (86–88). At UKM, compulsory elective postings of at least two weeks (international) or four weeks (local) provide students with opportunities for community-based service placements, offering them additional life skills and values. Such exposure may reduce cynicism and compassion fatigue while fostering resilience and fulfilment, particularly when learning goals emphasise meaningful service.

Our findings align with prior research showing that higher levels of professionalism and professional behaviour are associated with lower burnout scores and better mental health among medical students (89, 90). Professional values such as service, altruism and commitment to patients appear to buffer burnout by strengthening professional identity, enhancing meaning in work and promoting healthier coping and empathy (91, 92). Students who more fully internalise the values and role of “being a doctor” report lower levels of emotional exhaustion and cynicism and higher academic efficacy, suggesting that a clear, valued professional identity protects against burnout (93, 94). Professionalism framed around service, responsibility to society and care for the underserved can make demanding training feel purposeful, which is associated with lower burnout and better mental well-being (89). Higher professionalism and professional behaviour correlate with greater empathy, more positive coping strategies and less use of negative coping, which in turn are linked to lower burnout (95).

Conversely, students with higher burnout are more likely to report dishonest or unprofessional behaviours and less altruistic views about physicians’ responsibilities to society, indicating that erosion of professional values accompanies, and likely reinforces, burnout (92, 96). Greater engagement in values-based behaviour and less avoidance of personal values are associated with lower emotional exhaustion and depersonalisation (97).

### 4.6 Practical implications

Our findings carry important implications for medical education policy, particularly in Malaysia and other low- and middle-income contexts, highlighting the role of mental health educators in advancing students’ mental health. Institutions should implement structured mentor training to improve approachability and emotional intelligence, ensuring students feel supported. At UKM, outcomes could be strengthened by formalising peer mentorship as part of the curriculum, with measurable learning goals and structured mentor–mentee sessions.

Universities must also normalise mental health discussions, expand access to culturally sensitive counselling and create safe spaces that reduce stigma. Curriculum planners involving mental health educators, should adopt modular, flexible designs that promote autonomy, reduce stress and build intrinsic motivation.

Mental health educators can contribute by leading mentorship curriculum design, resilience workshops and mentor training in empathetic communication and distress recognition. They may also support the development of trauma-informed and gender-sensitive mentoring practices, as well as family-focused psychoeducation to realign parental expectations with student well-being. Ultimately, linking professional development with resilience will nurture of graduates who are not only clinically competent but also better equipped for long-term psychological health.

### 4.7 Limitations

This study has several limitations. First, it was conducted at a single institution, which restricts the generalisability of our findings to other medical schools and stages of training. In addition, the use of convenience sampling may limit the representativeness of the sample and generalisability of the results. Second, the peer mentorship programme examined was less structured than the formal lecturer mentorship at UKM, which may have affected its perceived effectiveness. Moreover, mentorship structures vary across Malaysian medical universities. For instance, at USM, the mentoring programme has evolved from involving all academic staff, to senior students, and now to volunteer lecturers (34). Such variability in mentor-mentee selection processes and programme design limits the applicability of our findings to other institutions. Third, our study relied on post-test questionnaires to evaluate the impact of peer mentorship on burnout, without pre-test assessments, which restricted our ability to measure changes over time. Furthermore, the cross-sectional design of the study prevents any causal inferences. Fourth, secondary assessments of related psychological constructs, such as anxiety, depression or PTSD symptoms, were not included, limiting the depth of mental health evaluation. Fifth, our study relied on self-reported questionnaires, which may be subject to social desirability or recall bias. Finally, the Persistence subscale from the SMMS-R demonstrated a low Cronbach’s alpha of 0.5 (45, 46), indicating limited internal consistency, which may have affected the reliability of findings related to motivation.

### 4.8 Future research

Future research should adopt longitudinal designs to better capture causal pathways and the sustained impact of interventions on burnout. Studies could evaluate curriculum-based interventions involving mental health educators, such as revisions to assessment strategies, structured reflective practices and early screening, to determine their effectiveness in strengthening resilience. Additionally, developing gender-sensitive and culturally grounded mentorship models may provide more tailored support, particularly in collectivist contexts such as Malaysia, and help identify which components of mentorship most effectively mitigate burnout.

## 5 Conclusion

Our study highlights several factors associated with burnout among medical students, including gender, parental occupation, mentorship quality and personal motivation and values. Burnout was more common among female students and those with parents in non-medical fields, reflecting the influence of societal and familial pressures on psychological well-being. Protective factors included approachable lecturers, supportive peer mentors, persistence in motivation, and strong service-oriented values. These findings emphasise the need for targeted interventions to support mentorship, motivation and professional values within medical training. Such strategies may help reduce academic disengagement and mitigate risks such as anxiety, depression and emotional exhaustion. Future research should examine the long-term effectiveness of these interventions and further investigate potential causal relationships, particularly the role of mental health educators in fostering resilience and safeguarding students’ mental health throughout their training.

## Data Availability

The raw data supporting the conclusions of this article will be made available by the authors, without undue reservation.
