# Supplementary material for: Burnout in medical students and its psychological correlates with mentorship, motivation and professional values
Source: Front Med (Lausanne). 2026 Feb 2;13:1752508. doi: 10.3389/fmed.2026.1752508 (PMC12907177; doi:10.3389/fmed.2026.1752508)
Supplement: Supplementary file 1 [file Data_Sheet_1.pdf]

Supplementary Material

Universiti Kebangsaan Malaysia (UKM) Mentorship Programmes

A) Lecturer mentorship

The lecturer mentorship programme at Universiti Kebangsaan Malaysia (UKM) pairs a faculty mentor with a small group of six to eight medical students with the aim to support the development of their personal growth and professional conduct. Medical students are given the options to choose a mentor of their preference, if available and agreeable by both parties. The programme is designed for mentors to provide feedback on medical students' progress regularly, with remediation when required. The programme also includes formative assessment of students' personal and professional attributes within the undergraduate medical curriculum.

Mentors are required to meet their student mentees regularly, at least once every semester (6 months). More frequent engagement is encouraged to address academic, personal or social concerns if needed, including stress management and emotional difficulties. During these meetings, mentors will review students' portfolios and reflective entries, provide constructive feedback and assess students' personal and professional conducts. Mentors will also attend scheduled mentor development and coordination sessions and participate in remedial processes for students requiring additional support.

To strengthen students' wellbeing, mentors will highlight potential early warning signs of burnout and psychological distress experienced by any individual student, and assist in establishing timely access to appropriate psychological support in accordance with institutional protocols.

B) Peer mentorship

The peer mentorship programme, commonly referred to as Buddy System, involves senior medical students providing mentorship to junior students. The programme traditionally focuses on the sharing of textbooks and notes as well as offering academic and clinical guidance. Unlike the lecturer mentorship programme, peer mentorship is less formally structured and largely depends on the initiative and commitment of individual mentors.

Buddy mentors are typically assigned during the second year of medical study. Second-year medical students will be paired with third-year students as peer mentors or buddies;

the same applies for fourth and fifth-year students. While the programme does not include formal mental health training or structured referral pathways, some mentors may encourage mentees to seek appropriate institutional or psychological support when symptoms suggestive of burnout or other psychiatric conditions are recognised.
